# Greater Body Fatness Is Associated With Higher Protein Expression of LEPR in Breast Tumor Tissues: A Cross-Sectional Analysis in the Women’s Circle of Health Study

**DOI:** 10.3389/fendo.2022.879164

**Published:** 2022-06-29

**Authors:** Adana A.M. Llanos, John B. Aremu, Ting-Yuan David Cheng, Wenjin Chen, Marina A. Chekmareva, Elizabeth M. Cespedes Feliciano, Bo Qin, Yong Lin, Coral Omene, Thaer Khoury, Chi-Chen Hong, Song Yao, Christine B. Ambrosone, Elisa V. Bandera, Kitaw Demissie

**Affiliations:** ^1^ Department of Epidemiology, Mailman School of Public Health, Columbia University Irving Medical Center, New York, NY, United States; ^2^ Herbert Irving Comprehensive Cancer Center, Columbia University Irving Medical Center, New York, NY, United States; ^3^ Department of Biostatistics and Epidemiology, Rutgers School of Public Health, Piscataway, NJ, United States; ^4^ Department of Epidemiology, University of Florida, Gainesville, FL, United States; ^5^ Department of Cancer Prevention and Control, Roswell Park Comprehensive Cancer Center, Buffalo, NY, United States; ^6^ Department of Pathology and Laboratory Medicine, Rutgers Robert Wood Johnson Medical School and Rutgers Cancer Institute of New Jersey, New Brunswick, NJ, United States; ^7^ Division of Research, Kaiser Permanente Northern California, Oakland, CA, United States; ^8^ Cancer Epidemiology and Health Outcomes, Rutgers Cancer Institute of New Jersey, New Brunswick, NJ, United States; ^9^ Department of Medicine, Rutgers Robert Wood Johnson Medical School and Rutgers Cancer Institute of New Jersey, New Brunswick, NJ, United States; ^10^ Department of Epidemiology and Biostatistics, SUNY Downstate Health Sciences University School of Public Health, Brooklyn, NY, United States

**Keywords:** adiposity, breast cancer, leptin (LEP), leptin receptor (LEPR), adiponectin (ADIPOQ), adiponectin receptors 1 and 2 (ADIPOR1, ADIPOR2), protein expression, gene expression

## Abstract

**Background:**

The mechanisms underlying the association of overall and central body fatness with poorer breast cancer outcomes remain unclear; altered gene and/or protein expression of the adipokines and their receptors in breast tumors might play a role.

**Methods:**

In a sample of Black and White women with primary invasive breast cancer, we investigated associations of body mass index (BMI), waist circumference, hip circumference, waist-to-hip ratio (WHR), fat mass index (FMI), and percent body fat with protein expression (log-transformed, n = 722) and gene expression (log2-transformed, n = 148) of leptin (LEP), leptin receptor (LEPR), adiponectin (ADIPOQ), and adiponectin receptors 1 and 2 (ADIPOR1, ADIPOR2). Multivariable linear models, adjusting for race, menopausal status, and estrogen receptor status, were used to assess these associations, with Bonferroni correction for multiple comparisons.

**Results:**

In multivariable models, we found that increasing BMI (β = 0.0529, 95% CI: 0.0151, 0.0906) and FMI (β = 0.0832, 95% CI: 0.0268, 0.1397) were associated with higher *LEP* gene expression, corresponding to 34.5% and 38.3% increases in *LEP* gene expression for a standard deviation (SD) increase in BMI and FMI, respectively. Increasing BMI (β = 0.0028, 95% CI: 0.0011, 0.0045), waist circumference (β = 0.0013, 95% CI: 0.0005, 0.0022), hip circumference (β = 0.0015, 95% CI: 0.0007, 0.0024), and FMI (β = 0.0041, 95% CI: 0.0015, 0.0067) were associated with higher LEPR protein expression. These associations equate to 16.8%, 17.6%, 17.7%, 17.2% increases in LEPR protein expression for a 1-SD increase in BMI, waist circumference, hip circumference, and FMI, respectively. Further, these associations were stronger among White and postmenopausal women and ER+ cases; formal tests of interaction yielded evidence of effect modification by race. No associations of body fatness with LEP protein expression, *LEPR* gene expression, or protein or gene expression of ADIPOQ, ADIPOR1, and ADIPOR2 were found.

**Conclusions:**

These findings support an association of increased body fatness – beyond overall body size measured using BMI – with higher *LEP* gene expression and higher LEPR protein expression in breast tumor tissues. Clarifying the impact of adiposity-related adipokine and adipokine receptor expression in breast tumors on long-term breast cancer outcomes is a critical next step.

## Introduction

Epidemiologic evidence suggests that increasing obesity, measured using body mass index (BMI), is associated with elevated risk of postmenopausal breast cancer (1, 2) and poorer breast cancer outcomes among both pre- and postmenopausal women (2–4). However, differences have been observed by estrogen receptor (ER) status (3, 5). While increased premenopausal obesity is associated with increased risk of ER- but not ER+ disease, postmenopausal obesity is similarly associated with increased risk of both ER- and ER+ disease (5, 6). On the other hand, increasing waist-to-hip ratio (WHR) is associated with increased risk of ER+ disease among premenopausal women and with increased risk of both ER+ and ER- disease among postmenopausal women (7).

While the molecular mechanisms underlying the impact of overall and central obesity on poorer breast cancer outcomes are not well understood, it has been hypothesized that the biological effects of the adipokines, adiponectin (ADIPOQ) and leptin (LEP), which are secreted by adipocytes (8–13), and their respective receptors (adiponectin receptors 1 and 2 [ADIPOR1, ADIPOR2] and leptin receptor [LEPR], respectively) might play a role. Further, exploration of the relationship between central adiposity (rather than overall body size as measured by BMI) and adipokines and adipokine receptors might be the missing link. Circulating ADIPOQ levels decrease with increasing BMI (14–16) and are associated with increased breast cancer risk (17–20). Conversely, circulating LEP levels increase with increasing BMI (21, 22) and are associated with increased breast cancer risk in some studies (17, 23–25). Less is known about adipokine receptor protein and gene expression levels in breast tumor tissues or their associations with more accurate and specific measures of body fatness derived from anthropometry (e.g., waist circumference, hip circumference, waist-to-hip ratio [WHR]) or from bioelectrical impedance analysis (BIA) (e.g., fat mass index [FMI], percent body fat [BF%]). These data might provide novel insights about the impact of body fatness and adiposity-related biomarker expression (at the tumor level) on breast cancer outcomes.

ADIPOQ is the most abundantly secreted adipokine by adipocytes (15, 26), and along with its receptors, is expressed in histologically normal and malignant breast tissues (27, 28). ADIPOQ has anti-inflammatory and anti-atherogenic properties (26, 29), inhibits cellular proliferation, and promotes apoptosis (10, 13, 30), implying a protective role in breast carcinogenesis. LEP, also secreted by adipocytes, is expressed in histologically normal and malignant breast cells, as is the LEPR (31, 32), LEP, once bound to LEPR, induces the activation of several signaling pathways, promotes cell growth and proliferation, and promotes angiogenesis (33–38).

Data from our prior research were the first to examine correlations between circulating ADIPOQ and LEP levels in plasma and levels within the breast, demonstrating that circulating adipokine levels are generally poor surrogates for levels within the local organ (39). More recently, we demonstrated that adipokine and adipokine receptor protein and gene expression in breast tumor tissues are associated with more aggressive tumor features associated with worse prognosis (40, 41). Specifically, lower LEPR protein expression was associated with ER- status, triple-negative (TN) subtype (40), while lower gene expression of *ADIPOQ*, *ADIPOR2*, *LEP*, and *LEPR* were associated with more aggressive breast tumor features, including higher tumor grade, larger tumor size, ER- status, and human epidermal growth factor receptor 2 (HER2)-enriched and TN subtypes (41).

In the current study, we hypothesize that measures of body fatness are associated with *LEPR*, *ADIPOR1*, and *ADIPOR2* expression profiles in the breast tumor microenvironment, which might contribute mechanistically to the development of more aggressive breast tumor phenotypes and poorer prognosis. To test this, we investigated associations of general obesity (BMI), body fat distribution (waist circumference, hip circumference, WHR), and body composition (FMI, BF%) with protein and gene expression of the adipokines and adipokine receptors in breast tissue specimens from participants in the Women’s Circle of Health Study (WCHS).

## Materials and Methods

### Study Sample and Data Collection

Study participants were women diagnosed with primary invasive breast cancer from 2001 through 2015 and enrolled in the WCHS (40, 41). Briefly, WCHS enrolled newly diagnosed breast cancer cases with histologically confirmed ductal carcinoma *in situ* (DCIS, stage 0) or invasive breast cancer (stages I–IV), who self-identified as either Black/African American or White, were 20–75 years of age, able to complete an interview in English, and had no history of cancer except non-melanomatous skin cancer. Data collection for the WCHS was conducted through in-person assessments (approximately 10 months after diagnosis) and included computer-assisted interviewer-administered questionnaires, as well as standardized protocols for taking anthropometric measurements during a home visit, including height, weight, waist circumference, and hip circumference, and body composition using a portable BIA scale (42). The baseline interview ascertained information on sociodemographic factors as well as established or probable breast cancer risk factors, including family and personal health history, reproductive history, hormone therapy use, and lifestyle exposures.

Nearly all WCHS participants (98%) consented to medical records release and for these participants, medical and pathology records were requested and retrieved from providers and institutions where participants reported receiving breast cancer care. Relevant clinical and breast tumor clinicopathologic data were abstracted and entered in an electronic database (43, 44).

### Collection of Archived Breast Tumor Specimens and Tissue Microarray Construction

Tumor blocks and/or slides for WCHS participants were retrieved from hospitals upon written consent, with a retrieval rate of approximately 85%. Upon receipt at the Data Bank and Biorepository (DBBR) at Roswell Park Comprehensive Cancer Center, a board-certified pathologist (TK) reviewed hematoxylin and eosin (H&E) slides and circled areas where cores were selected for tissue microarray (TMA) construction. TMA cores ranged in size from 0.6 mm to 1.2 mm in diameter, and most WCHS participants’ tumors were represented by at least two TMA cores (range: 1 to 6 cores), which were placed into a recipient formalin-fixed paraffin-embedded (FFPE) block. The location of each core was recorded in a detailed TMA map file. The completed TMAs were stored at room temperature.

### Protein Expression Analysis

For each WCHS participant included in the protein expression analysis (n = 722), immunohistochemistry (IHC) was used to stain TMAs of breast tumor specimens for LEP, LEPR, ADIPOQ, ADIPOR1, and ADIPOR2 as previously described (40). Briefly, IHC staining was performed using Ventana Discovery XT Automated Slide Stainer (Ventana Medical Systems, Inc., Tucson, AZ, USA). Deparaffinization, antigen retrieval, blocking, DAB detection, counterstain, post-counterstain, and slide cleaning steps were automated on the Discovery XT. Primary antibodies and secondary antibodies were manually applied at programmed steps. The following primary antibodies were used: rabbit monoclonal OB (LEP) antibody (1:40 dilution; Santa Cruz, cat #sc-842), mouse monoclonal Ob-R (LEPR) antibody (1:25 dilution; Santa Cruz, cat #sc-8391), mouse monoclonal adiponectin antibody (1:30 dilution; Abcam, cat #ab22554), rabbit monoclonal ADIPOR1 antibody (1:350 dilution; Abcam, cat #ab126611), and goat polyclonal ADIPOR2 antibody (1:25 dilution; Abcam, cat #ab77612). Optimal staining on control slides (human breast tissue TMAs) was obtained for each individual antibody. IHC was performed using the optimized conditions on the experimental WCHS TMA slides as well as on additional control slides. Primary antibodies were incubated at 37°C for 1–2 h; secondary antibodies were incubated at 37°C for 1 h, followed by either the DAB Map Detection Kit (Ventana, 760-124) or ChromoMap DAB kit (Ventana, 760-159). Slides were counterstained with hematoxylin (Ventana, 760-2021)and bluing reagent (Ventana, 760-2037) before cover slipping. A digital pathology analysis platform (VisioPharm, Hoersholm Denmark) was used to quantify protein expression of the adipokine receptors on each tissue core (45). Quantitative results were reported as a protein expression score defined as effective staining intensity (ESI) within the effective staining area (ESA) (45). Specimen artifacts, such as tissue folding were manually excluded from quantification. A board-certified pathologist (MAC) semi-quantitatively evaluated IHC expression for each tissue core stained (45). Semi-quantitative expression results were reported as: 0 (negative), 1 (weak expression), 2 (moderate expression), or 3 (strong expression). We observed high concordance between unsupervised, quantitative scores and pathologist-generated, semi-quantitative scores for LEP (r = 0.70, *P*<0.0001) and LEPR (r = 0.71, *P<*0.0001) (40). In the present analysis we included only quantitative protein expression data for LEP, LEPR, ADIPOQ, ADIPOR1, and ADIPOR2, which were averaged for participants with multiple TMA cores. Log-transformed protein expression data were used in the subsequent analysis.

### Gene Expression Analysis

For each WCHS participant included in the gene expression analysis (n = 148), RNA was extracted from two 10µm curls (from representative breast tumor blocks without any pre-selection based on either the tumor or stromal contents so as to maintain and capture the entire tumor lesion and surrounding microenvironment) using the High Pure FFPET RNA Isolation Kit (Roche Molecular Systems, Inc., Pleasanton, CA, USA) and quantified using Qubit and Agilent Bioanalyzer (Agilent Technologies, Santa Clara, CA, USA). Gene expression of *ADIPOQ, ADIPOR1*, *ADIPOR2*, LEP, and *LEPR* were quantitated using NanoString nCounter^®^ technology (NanoString Technologies, Seattle, WA, USA) (41). Raw count data were subjected to a series of normalization steps, including positive controls, housekeeping genes, and background subtraction, and the normalized data were log2-transformed and used in subsequent analyses (41).

### Statistical Analyses

Descriptive statistics (mean and standard deviation [SD] and frequency and proportions) were used to describe the study sample and Pearson’s correlation analysis was used to assess pairwise correlations between adipokine receptor protein and gene expression. Multivariable linear regression models were utilized to evaluate the associations of BMI, waist circumference, hip circumference, WHR, FMI, and BF% with protein and gene expression of the adipokines and adipokine receptors. The difference in protein and gene expression per increase in SD of body fatness measures was also estimated, and a percentage increase was estimated as [exp(beta) - 1] x 100%. Models were adjusted for age, race, menopausal status, and ER status. All reported *P*-values are two-sided and *P*<0.05 was considered statistically significant. To account for multiple comparisons, we used Bonferroni correction with a criterion of *P*<0.0083 (i.e., 0.05/6) for statistical significance, given that there were six tests of association for protein and gene expression of each marker of interest. Analyses were performed using STATA (version 17, StataCorp, College Station, TX).

## Results

Sociodemographic and tumor characteristics of the study sample included in the protein expression and the gene expression analytic samples are shown in **Table 1**. Across both groups, most participants met the criteria for increased metabolic risk (46, 47) based on overall obesity (BMI >30 kg/m^2^ [50.6%]), central obesity (waist circumference >88 cm [74.4%] and/or WHR >0.85 [64.1%]), elevated/abnormal FMI (≥9.5 kg/m^2^ [73.4%]), and BF% (>35% [76.2%]).

**Table 1:** Select characteristics of analytic samples included in the adipokine receptor protein expression and gene expression analysis.

	Protein expression, N = 722^a^	Gene expression, N = 148^b^
*Sociodemographic and clinical characteristics*	n (%)	n (%)
Age at diagnosis (years), mean ± SD	52.58 ± 10.83	53.08 ± 10.34
Menopausal status		
Premenopausal	325 (46.43)	68 (47.22)
Postmenopausal	375 (53.57)	76 (52.78)
Race		
Black/African American	541 (77.29)	109 (75.69)
White	159 (22.71)	35 (24.31)
Body mass index (kg/m^2^), mean ± SD	30.72 ± 6.99	30.89 ± 7.50
Waist circumference (cm), mean ± SD	98.63 ± 15.47	99.15 ± 15.66
Hip circumference (cm), mean ± SD	112.13 ± 13.30	111.86 ± 13.88
Waist-to-hip ratio, mean ± SD	0.87 ± 0.08	0.88 ± 0.07
Fat mass index, mean ± SD	12.44 ± 4.82	12.61 ± 5.27
Percent body fat (%),mean ± SD	39.30 ± 7.77	39.20 ± 8.07
*Breast tumor characteristics*		
Tumor grade		
Well differentiated	107 (16.85)	13 (9.03)
Moderately differentiated	219 (34.49)	45 (31.25)
Poorly differentiated	309 (48.66)	86 (59.72)
Tumor size		
<1.0 cm	149 (20.64)	22 (14.86)
1.0-2.0 cm	281 (38.92)	64 (43.24)
>2.0 cm	292 (40.44)	62 (41.89)
AJCC stage		
Stage 0	62 (8.96)	1 (0.72)
Stage I	257 (37.14)	54 (39.13)
Stage II	271 (39.16)	66 (47.83)
Stage III	96 (13.87)	14 (10.14)
Stage IV	6 (0.87)	3 (2.17)
ER status		
ER+	505 (70.14)	84 (56.76)
ER-	215 (29.86)	64 (43.24)
HER2 status		
HER2-	412 (81.58)	112 (75.68)
HER2+	93 (18.42)	36 (24.32)

a In the protein expression sample, age was missing for 22 (3%); BMI was missing for 23 (3.2%); waist circumference, hip circumference, and waist-to-hip ratio were missing for 33 (4.6%); fat mass index was missing for 66 (9.1%); percent body fat was missing for 64 (8.9%); tumor grade was missing for 87 (12%); tumor stage was missing for 30 (4.2%); ER status was missing for 2 (0.3%), and HER2 status was missing for 18 (2.5%) participants.

b In the gene expression sample, age, menopausal status, race, and BMI was missing for 4 (2.7%) participants; waist circumference, hip circumference, and waist-to-hip ratio were missing for 5 (3.4%) participants; fat mass index and percent body fat were missing for 12 (8.1%) participants; tumor grade was missing for 4 (2.7%) participants; and tumor stage was missing for 10 (6.8%) participants.

There was no significant correlation between LEP protein and *LEP* gene expression (r = 0.11, P = 0.21), a weak positive correlation between LEPR protein and *LEPR* gene expression (r = 0.29, P = 0.0006), a weak positive correlation between ADIPOQ protein and *ADIPOQ* gene expression (r = 0.18, P = 0.04), no significant correlation between ADIPOR1 protein and *ADIPOR1* gene expression (r = -0.03, P = 0.69), and a very weak positive correlation between ADIPOR2 protein and *ADIPOR2* gene expression (r = 0.18, P = 0.04). In models adjusting for age, race, menopausal status, and ER status, we found that there were no significant associations between measures of body fatness and LEP protein expression (**Table 2**). In contrast, we found that women with increasing BMI (β = 0.0529, 95% CI: 0.0151, 0.0906), waist circumference (β = 0.0247, 95% CI: 0.0065, 0.0429), hip circumference (β = 0.0259, 95% CI: 0.0066, 0.0453), FMI (β = 0.0832, 95% CI: 0.0268, 0.1397), and BF% (β = 0.0396, 95% CI: 0.0025, 0.0767) had higher *LEP* gene expression. Only the findings for BMI and FMI were significant with correction for multiple comparisons and corresponds to 34.5% and 38.3% increases in *LEP* gene expression for a 1-SD increase in BMI and FMI, respectively. Women with greater body fatness had significantly higher LEPR protein expression: BMI (β = 0.0028, 95% CI: 0.0011, 0.0045), waist circumference (β = 0.0013, 95% CI: 0.0005, 0.0022), hip circumference (β = 0.0015, 95% CI: 0.0007, 0.0024), and FMI (β = 0.0041, 95% CI: 0.0015, 0.0067) (**Table 3**). These associations equate to 16.8%, 17.6%, 17.7%, 17.2% increases in LEPR protein expression for a 1-SD increase in BMI, waist circumference, hip circumference, and FMI, respectively. WHR was not associated with LEPR protein expression. Upon further adjustment for waist circumference, the observed associations between BMI (P = 0.08), hip circumference (P = 0.13), and BF% (P = 0.26) were consistent but attenuated, while the association for FMI was slightly stronger (β = 0.0055, 95% CI: 0.0005, 0.0010; 24.1% increase in LEPR protein expression), although not statistically significant (data not shown). Conversely, we found no association between body fatness and *LEPR* gene expression. Associations between body fatness and ADIPOQ expression (**Table 4**), ADIPOR1 expression (**Table 5**) and ADIPOR2 expression (**Table 6**) were not statistically significant, but the coefficients suggested that increasing body fatness might be associated with lower protein expression of ADIPOQ, ADIPOR1, and ADIPOR2, lower *ADIPOQ* gene expression. and higher gene expression of *ADIPOR1* and *ADIPOR2*.

**Table 2:** Multivariable-adjusted associations of body fatness measures with LEP protein and *LEP* gene expression in breast tumor tissues.

	LEP protein expression				*LEP* gene expression			
	n	β (95% CI)	β_standardized_	*P*	n	β (95% CI)	β_standardized_	*P*
Body mass index (kg/m^2^)	635	0.0013 (-0.0007, 0.0033)	0.0650	0.189	144	0.0529 (0.0151, 0.0906)	0.2962	0.007**
Waist circumference (cm)	624	0.0003 (-0.0006, 0.0013)	0.0338	0.507	143	0.0247 (0.0065, 0.0429)	0.2880	0.009*
Hip circumference (cm)	624	0.0006 (-0.0004, 0.0016)	0.0570	0.235	143	0.0259 (0.0066, 0.0453)	0.2684	0.01*
Waist-to-hip ratio	624	-0.0606 (-0.2503, 0.1291)	-0.0328	0.532	143	1.5986 (-2.5554, 5.7526)	0.0880	0.452
Fat mass index (kg/m^2^)	594	0.0012 (-0.0019, 0.0044)	0.0405	0.440	136	0.0832 (0.0268, 0.1397)	0.3245	0.005**
Percent body fat (%)	596	-0.0002 (-0.0021, 0.0018)	-0.0103	0.843	136	0.0396 (0.0025, 0.0767)	0.2359	0.038*

Protein expression scores reflect quantitative protein expression (using immunohistochemistry) of LEP as analyzed through an automated/unsupervised scoring (quantitative) methodology. The scores estimate the effective staining intensity (ESI) within the effective staining area (ESA) of the biomarker in question (mean±SD of log-transformed values are shown). Gene expression scores reflect normalized, log2-transformed gene expression of LEP as analyzed through the Nanostring nCounter Analysis System. Each model was generated using multiple linear regression adjusting for age, race, menopausal status, and estrogen receptor status.

*Statistically significant at P<0.05; **Statistically significant with correction for multiple comparisons (P <0.0083).

**Table 3:** Multivariable-adjusted associations of body fatness measures with LEPR protein and LEPR gene expression in breast tumor tissues.

	LEPR protein expression				*LEPR* gene expression			
	n	β (95% CI)	β_standardized_	*P*	n	β (95% CI)	β_standardized_	*P*
Body mass index (kg/m^2^)	571	0.0028 (0.0011, 0.0045)	0.155	0.002**	144	0.0062 (-0.0187, 0.0312)	0.053	0.625
Waist circumference (cm)	561	0.0013 (0.0005, 0.0022)	0.162	0.001**	143	-0.0003 (-0.0122, 0.0116)	-0.005	0.962
Hip circumference (cm)	561	0.0015 (0.0007, 0.0024)	0.163	0.001**	143	-0.0006 (-0.0134, 0.0122)	-0.009	0.927
Waist-to-hip ratio	561	0.0537 (-0.1131, 0.2206)	0.033	0.528	143	-0.1725 (-2.8365, 2.4914)	-0.014	0.899
Fat mass index (kg/m^2^)	534	0.0041 (0.0015, 0.0067)	0.159	0.002**	136	0.0091 (-0.0275, 0.0458)	0.055	0.626
Percent body fat (%)	536	0.0020 (0.0004, 0.0036)	0.124	0.016*	136	-0.0012 (-0.0246, 0.0228)	-0.008	0.941

Protein expression scores reflect quantitative protein expression (using immunohistochemistry) of LEPR as analyzed through an automated/unsupervised scoring (quantitative) methodology. The scores estimate the effective staining intensity (ESI) within the effective staining area (ESA) of the biomarker in question (mean±SD of log-transformed values are shown). Gene expression scores reflect normalized, log2-transformed gene expression of LEPR as analyzed through the Nanostring nCounter Analysis System. Each model was generated using multiple linear regression adjusting for age, race, menopausal status, and estrogen receptor status.

*Statistically significant at P<0.05; **Statistically significant with correction for multiple comparisons (P <0.0083).

**Table 4:** Multivariable-adjusted associations of body fatness measures with ADIPOQ protein and *ADIPOQ* gene expression in breast tumor tissues.

	ADIPOQ protein expression				*ADIPOQ* gene expression			
	n	β (95% CI)	β_standardized_	*P*	n	β (95% CI)	β_standardized_	*P*
Body mass index (kg/m^2^)	618	0.0009 (-0.0013, 0.0032)	0.0405	0.418	144	0.0036 (-0.0515, 0.0587)	0.0140	0.897
Waist circumference (cm)	608	0.0008 (-0.0003, 0.0019)	0.0765	0.137	143	-0.0065 (-0.0328, 0.0198)	-0.0518	0.629
Hip circumference (cm)	608	0.0008 (-0.0004, 0.0019)	0.0654	0.176	143	0.0041 (-0.0241, 0.0323)	0.0291	0.775
Waist-to-hip ratio	608	0.0840 (-0.1239, 0.2919)	0.0414	0.429	143	-5.1582 (-10.9547, 0.6382)	-0.1943	0.083
Fat mass index (kg/m^2^)	583	0.0019 (-0.0016, 0.0053)	0.0565	0.285	136	-0.0060 (-0.0890, 0.0770)	-0.0160	0.888
Percent body fat (%)	584	0.0006 (-0.0015, 0.0027)	0.0281	0.589	136	-0.0280 (-0.813, 0.0254)	-0.1144	0.306

Protein expression scores reflect quantitative protein expression (using immunohistochemistry) of ADIPOQ as analyzed through an automated/unsupervised scoring (quantitative) methodology. The scores estimate the effective staining intensity (ESI) within the effective staining area (ESA) of the biomarker in question (mean±SD of log-transformed values are shown). Gene expression scores reflect normalized, log2-transformed gene expression of ADIPOQ as analyzed through the Nanostring nCounter Analysis System. Each model was generated using multiple linear regression adjusting for age, race, menopausal status, and estrogen receptor status. *Statistically significant at P<0.05; **Statistically significant with correction for multiple comparisons (P <0.0083).

**Table 5:** Multivariable-adjusted associations of body fatness measures with ADIPOR1 protein and ADIPOR1 gene expression in breast tumor tissues.

	ADIPOR1 protein expression				*ADIPOR1* gene expression			
	n	β (95% CI)	β_standardized_	*P*	n	β (95% CI)	β_standardized_	*P*
Body mass index (kg/m^2^)	665	-0.0008 (-0.0028, 0.0013)	-0.035	0.473	144	0.0070 (-0.0045, 0.0184)	0.128	0.234
Waist circumference (cm)	654	-0.0002 (-0.0011, 0.0008)	-0.015	0.761	143	0.0048 (-0.0007, 0.0102)	0.181	0.090
Hip circumference (cm)	654	-0.0004 (-0.0014, 0.0007)	-0.032	0.497	143	0.0032 (-0.0032, 0.0086)	0.091	0.367
Waist-to-hip ratio	654	0.0474 (-0.1447, 0.2394)	0.024	0.629	143	1.1785 (-0.0507, 2.4078)	0.212	0.062
Fat mass index (kg/m^2^)	624	-0.0019 (-0.0050, 0.0013)	-0.059	0.249	136	0.0081 (-0.0091, 0.0252)	0.104	0.357
Percent body fat (%)	626	-0.0016 (-0.0036, 0.0003)	-0.083	0.099	136	0.0045 (-0.0066, 0.0156)	0.090	0.424

Protein expression scores reflect quantitative protein expression (using immunohistochemistry) of ADIPOR1 as analyzed through an automated/unsupervised scoring (quantitative) methodology. The scores estimate the effective staining intensity (ESI) within the effective staining area (ESA) of the biomarker in question (mean±SD of log-transformed values are shown). Gene expression scores reflect normalized, log2-transformed gene expression of ADIPOR1 as analyzed through the Nanostring nCounter Analysis System. Each model was generated using multiple linear regression adjusting for age, race, menopausal status, and estrogen receptor status.

**Table 6:** Multivariable-adjusted associations of body fatness measures with ADIPOR2 protein and ADIPOR2 gene expression in breast tumor tissues.

	ADIPOR2 protein expression				*ADIPOR2* gene expression			
	n	β (95% CI)	β_standardized_	*P*	n	β (95% CI)	β_standardized_	*P*
Body mass index (kg/m^2^)	584	-0.0003 (-0.0028, 0.0023)	-0.010	0.844	144	0.0098 (-0.0103, 0.0300)	0.100	0.346
Waist circumference (cm)	575	-0.0003 (-0.0015, 0.0009)	-0.029	0.585	143	0.0085 (-0.0012, 0.0182)	0.182	0.088
Hip circumference (cm)	575	-0.0001 (-0.0014, 0.0012)	-0.008	0.873	143	0.0046 (-0.0058, 0.0150)	0.087	0.387
Waist-to-hip ratio	575	-0.0389 (-0.2773, 0.1995)	-0.017	0.749	143	2.1901 (0.0181, 4.3621)	0.221	0.050
Fat mass index (kg/m^2^)	552	0.0002 (-0.0037, 0.0040)	0.004	0.939	136	0.0155 (-0.0146, 0.0456)	0.113	0.313
Percent body fat (%)	554	0.0007 (-0.0017, 0.0031)	0.031	0.557	136	0.0136 (-0.0058, 0.0330)	0.151	0.171

Protein expression scores reflect quantitative protein expression (using immunohistochemistry) of ADIPOR2 as analyzed through an automated/unsupervised scoring (quantitative) methodology. The scores estimate the effective staining intensity (ESI) within the effective staining area (ESA) of the biomarker in question (mean±SD of log-transformed values are shown). Gene expression scores reflect normalized, log2-transformed gene expression of ADIPOR2 as analyzed through the Nanostring nCounter Analysis System. Each model was generated using multiple linear regression adjusting for age, race, menopausal status, and estrogen receptor status.

Given the multivariable-adjusted associations observed between measures of body fatness and LEPR protein expression levels, we explored potential differences by race (**Table 7**), menopausal status (**Table 8**), and ER status (**Table 9**). Qualitatively, our observation that increasing body fatness measures are associated with higher LEPR protein expression appeared stronger among White women, postmenopausal women, and ER+ cases. Formal tests of interaction yielded statistically significant evidence of effect modification by race for some body fatness measures (BMI, P = 0.041; FMI, P = 0.016; and BF%, P = 0.019), but not others (waist circumference, P = 0.080; hip circumference, P = 0.086) (data not shown). However, we observed no evidence of effect modification by menopausal status (P-values for all body fatness measures >0.05), and limited evidence of effect modification by ER status (BMI, P = 0.318; waist circumference, P = 0.093; hip circumference, P = 0.059; WHR, P = 0.821; FMI, P = 0.250; and BF%, P = 0.553) (data not shown).

**Table 7:** Multivariable-adjusted associations of body fatness measures with LEPR protein expression in breast tumor tissues, stratified by race.

Black women	LEPR protein expression			
	n	β (95% CI)	β_standardized_	*P*
Body mass index (kg/m^2^)	455	0.0016 (0.0000, 0.0031)	0.0906	0.049*
Waist circumference (cm)	445	0.0006 (-0.0001, 0.0014)	0.0782	0.101
Hip circumference (cm)	445	0.0007 (-0.0001, 0.0015)	0.0791	0.089
Waist-to-hip ratio	445	0.0267 (-0.1276, 0.1810)	0.0169	0.735
Fat mass index (kg/m^2^)	428	0.0019 (-0.0042, 0.0042)	0.0791	0.100
Percent body fat (%)	428	0.0008 (-0.0007, 0.0023)	0.0501	0.302
**White women**	**LEPR protein expression**			
	**n**	**β (95% CI)**	**β_standardized_**	***P***
Body mass index (kg/m^2^)	116	0.0050 (0.0011, 0.0089)	0.2303	0.014*
Waist circumference (cm)	116	0.0020 (0.0003, 0.0037)	0.2315	0.022*
Hip circumference (cm)	116	0.0021 (0.0003, 0.0040)	0.2055	0.027*
Waist-to-hip ratio	116	0.1634 (-0.2107, 0.5376)	0.0812	0.394
Fat mass index (kg/m^2^)	106	0.0078 (0.0016, 0.0139)	0.2371	0.015*
Percent body fat (%)	108	0.0043 (0.0008, 0.0078)	0.2292	0.018*

Protein expression scores reflect quantitative protein expression (using immunohistochemistry) of LEPR as analyzed through an automated/unsupervised scoring (quantitative) methodology. The scores estimate the effective staining intensity (ESI) within the effective staining area (ESA) of the biomarker in question (mean±SD of log-transformed values are shown). Each model was generated using multiple linear regression adjusting for age, menopausal status, and ER status. *Statistically significant at P<0.05.

**Table 8:** Multivariable-adjusted associations of body fatness measures with LEPR protein expression in breast tumor tissues, stratified by menopausal status.

Premenopausal women	LEPR protein expression			
	n	β (95% CI)	β_standardized_	*P*
Body mass index (kg/m^2^)	264	0.0019 (-0.0002, 0.0040)	0.1090	0.078
Waist circumference (cm)	261	0.0009 (-0.0001, 0.0019)	0.1033	0.096
Hip circumference (cm)	261	0.0007 (-0.0004, 0.0018)	0.0728	0.231
Waist-to-hip ratio	261	0.1446 (-0.0755, 0.3647)	0.0818	0.199
Fat mass index (kg/m^2^)	247	0.0026 (-0.0006, 0.0058)	0.1023	0.111
Percent body fat (%)	247	0.0014 (-0.0007, 0.0034)	0.0854	0.183
**Postmenopausal women**	**LEPR protein expression**			
	**n**	**β (95% CI)**	**β_standardized_**	***P***
Body mass index (kg/m^2^)	307	0.0027 (0.0007, 0.0048)	0.1483	0.010*
Waist circumference (cm)	300	0.0010 (0.0001, 0.0020)	0.1244	0.033*
Hip circumference (cm)	300	0.0015 (0.0004, 0.0025)	0.1575	0.005**
Waist-to-hip ratio	300	-0.0420 (-0.2321, 0.1480)	-0.0259	0.665
Fat mass index (kg/m^2^)	287	0.0039 (0.0008, 0.0069)	0.1489	0.013*
Percent body fat (%)	289	0.0021 (0.0001, 0.0040)	0.1262	0.036*

Protein expression scores reflect quantitative protein expression (using immunohistochemistry) of LEPR as analyzed through an automated/unsupervised scoring (quantitative) methodology. The scores estimate the effective staining intensity (ESI) within the effective staining area (ESA) of the biomarker in question (mean±SD of log-transformed values are shown). Each model was generated using multiple linear regression adjusting for age, race, and ER status.

*Statistically significant at P<0.05; **Statistically significant with correction for multiple comparisons (P <0.0083).

**Table 9:** Multivariable-adjusted associations of body fatness measures with LEPR protein expression in breast tumor tissues, stratified by ER status.

ER+ cases	LEPR protein expression			
	n	β (95% CI)	β_standardized_	*P*
Body mass index (kg/m^2^)	405	0.0028 (0.0011, 0.0045)	0.1620	0.001**
Waist circumference (cm)	399	0.0014 (0.0006, 0.0022)	0.1712	0.001**
Hip circumference (cm)	399	0.0015 (0.0007, 0.0024)	0.1717	<0.0001**
Waist-to-hip ratio	399	0.0540 (-0.1133, 0.2213)	0.0342	0.527
Fat mass index (kg/m^2^)	382	0.0041 (0.0015, 0.0067)	0.1610	0.002**
Percent body fat (%)	385	0.0020 (0.0004, 0.0037)	0.1267	0.017*
**ER- cases**	**LEPR protein expression**			
	**n**	**β (95% CI)**	**β_standardized_**	***P***
Body mass index (kg/m^2^)	166	0.0011 (-0.0018, 0.0040)	0.0613	0.440
Waist circumference (cm)	162	0.0001 (-0.0013, 0.0014)	0.0047	0.954
Hip circumference (cm)	162	-0.0001 (-0.0017, 0.0015)	-0.0105	0.894
Waist-to-hip ratio	162	0.0221 (-0.2635, 0.3076)	0.0132	0.880
Fat mass index (kg/m^2^)	152	0.0013 (-0.0026, 0.0053)	0.0546	0.510
Percent body fat (%)	151	0.0011 (-0.0016, 0.0037)	0.0654	0.432

Protein expression scores reflect quantitative protein expression (using immunohistochemistry) of LEPR as analyzed through an automated/unsupervised scoring (quantitative) methodology. The scores estimate the effective staining intensity (ESI) within the effective staining area (ESA) of the biomarker in question (mean±SD of log-transformed values are shown). Each model was generated using multiple linear regression adjusting for age, race, and menopausal status.

*Statistically significant at P<0.05; **Statistically significant with correction for multiple comparisons (P <0.0083).

## Discussion

Building on our prior research, here we examined the associations of body fatness measures with protein and gene expression of the adipokines, LEP and ADIPOQ, and the adipokine receptors, LEPR, ADIPOR1, and ADIPOR2 in breast tumor tissues. To our knowledge, this is the first study to investigate these associations in women with breast cancer. Partially consistent with our hypothesis, we found that greater body fatness is associated with increased *LEP* gene expression and LEPR protein expression, although we observed no associations between body fatness and *LEPR* gene expression, nor with protein or gene expression of ADIPOQ, ADIPOR1, and ADIPOR2.

Past studies show that BMI is positively associated with circulating leptin concentrations and inversely associated with circulating adipokine concentrations, which are associated with increased risk of some obesity-related cancers including breast cancer (reviewed by Yoon et al. (48)). Our finding that increasing measures of body fatness are positively associated with LEPR protein expression in breast tumors independent of age and menopausal status (with correction for multiplicity) support the hypothesis that LEPR protein expression in breast tumor tissues plays a role in breast carcinogenesis (49–54). Interestingly, our analysis showed significant effect modification by race (stronger among White women) and marginally significant effect modification by ER status (suggestion of stronger associations among ER+ cases, although our analysis was underpowered given the small sample of ER- cases). These findings further highlight the complex interplay among LEPR protein expression, adiposity, race, and breast tumor phenotype (55–58), which might require more precise adiposity measures, and identification and refinement of adiposity-associated biomarkers within breast tumor tissues that can predict breast cancer outcomes. We observed no significant associations between body fatness and *LEPR* gene expression, but we previously showed that gene expression of *LEPR* is significantly lower in ER- and TN breast tumors relative to ER+ and luminal A subtypes, respectively (41). While our sample with data on adipokine receptor gene expression was small and limited our statistical power, larger studies in the future will help clarify these findings. Nonetheless, our findings suggest that distribution of adiposity and adiposity-related expression profiles of the adipokines and adipokine receptors, especially LEP and LEPR, in the local organ might have differential impacts on breast cancer based on tumor subtype, and the crosstalk between ER and adipokine biomarkers and other inflammatory biomarkers might play a role (59).

Prior analysis from WCHS reported a lack of association between BMI and breast cancer risk, but higher hip circumference and waist circumference were associated with more than 2-fold increased risk of pre-menopausal breast cancer among women in the fourth quartiles for each measure compared to the first quartile.^42^ Further, findings from WCHS also showed that compared to BMI, WHR was more strongly associated with overall and breast cancer-specific mortality among Black women. Specifically, compared to the first quartile, women in the fourth quartile of WHR had 61% and 68% increased risk of overall and breast cancer specific death, respectively, while women with class I and class II obesity (compared to normal weight) had statistically non-significant increased risk of death ranging from 17—33% (44). From the combination of these findings, investigations of the associations between more accurate measures of adiposity and adipose tissue distribution (including overall adiposity, visceral adiposity, and subcutaneous adiposity assessed through computed tomography [CT]), in association with adipokine receptor protein and gene expression are critical to elucidating the impact of adiposity on breast carcinogenesis and progression. Such data are critical to determining the clinical utility of adipokine receptor expression profiles as biomarkers of breast cancer risk and prognosis and might contribute to the development of novel interventions and therapeutics targeting high breast tumor expression of these markers, particularly of LEPR, as a means of improving outcomes.

An important strength of this study is that it adds new knowledge regarding the potential impact of overall and central body fatness on adiposity-related biomarkers in breast tumor tissues. Our findings suggest that measures of body fatness are associated with the expression of adipokine receptors – primarily LEP and LEPR – in breast tumors. From this, we generated new hypotheses about the mechanisms linking central adiposity with breast cancer outcomes, which will be pursued. Another strength was the opportunity to perform stratified analysis of the associations of interest by race, menopausal status, and ER status, yielding novel findings. Lastly, our population-based sample that included a large proportion of Black women with breast cancer was also a strength. This study also has some limitations worth noting, including a relatively small sample size (particularly in the gene expression analysis [n = 148]), which may have reduced the power to detect meaningful associations and limit our ability to fully evaluate the complex associations of body fatness with breast cancer. Relatedly, our analysis included multiple comparisons which may have increased the likelihood of observing statistically significant associations. However, we addressed this concern using Bonferroni correction.

Despite these limitations, the findings substantiated our hypothesis that measures of body fatness are associated with expression of adipokine biomarkers in breast tumors. These data are an important step towards understanding the biologic effects of and potential mechanisms linking adiposity with breast cancer risk and prognosis.

## Data Availability Statement

The raw data supporting the conclusions of this article will be made available by the authors, without undue reservation.

## Ethics Statement

The studies involving human participants were reviewed and approved by Rutgers University Institutional Review Board. The patients/participants provided their written informed consent to participate in this study.

## Author Contributions

AL: Grant funding, study conception and design, data collection, data analysis, data interpretation, and writing. JA: Literature search and data interpretation. T-YC: Data interpretation and manuscript editing. WC: Data collection and manuscript editing. MC: Pathology review, data collection, and manuscript editing. EC: Data interpretation and manuscript editing. BQ: Data collection, data interpretation, and manuscript editing. YL: Data analysis, data interpretation, and manuscript editing. CO: Data interpretation and manuscript editing. TK: Pathology review, data collection, and manuscript editing. C-CH: Grant funding, data collection, and manuscript editing. SY: Data collection, data interpretation, and manuscript editing. CA: Grant funding, data collection, data interpretation, and manuscript editing. EB: Grant funding, data collection, data interpretation, and manuscript editing. KD: Grant funding, data collection, data interpretation, and manuscript editing. All authors read and approved the final manuscript. All authors contributed to the article and approved the submitted version.

## Funding

This study was supported by funding from the National Cancer Institute of the National Institutes of Health under the following award numbers: K01CA193527 (awarded to AL), P01CA151135 (awarded to CA), P30CA072720 (awarded to S. Libutti), R01CA100598 (awarded to CA), R01CA185623 (awarded to EB, KD, and C-CH), K08CA172722 (awarded to CO), K07CA201334 (awarded to T-YC), and K01CA226155 (awarded to EC). Support was also received by the U.S. Army Medical Research and Development Command under award number DAMD‐17‐01‐1‐0334 (awarded to D.H. Bovbjerg), the Breast Cancer Research Foundation (awarded to CA and C-CH), and a gift from the Philip L. Hubbell Family (awarded to CA). Tumor samples were received, processed and tracked under the auspices of the Roswell Park Comprehensive Cancer Center Data Bank and BioRepository Shared Resource, with funding from NCI-CCSG P30CA16056. Services, results and/or products in support of this research project were generated using the Rutgers Cancer Institute of New Jersey Biomedical Informatics Shared Resource (P30CA072720-5917) and the Biospecimen Repository and Histopathology Service Shared Resource (P30CA072720-5919). The New Jersey State Cancer Registry is funded by the National Cancer Institute’s Surveillance, Epidemiology and End Results (SEER) Program (#75N91021D00009), Centers for Disease Control and Prevention’s National Program of Cancer Registries (#5NU58DP006279) with additional support from the State of New Jersey and the Rutgers Cancer Institute of New Jersey.

## Conflict of Interest

Author EC is employed by the company Kaiser Permanente Northern California.

The remaining authors declare that the research was conducted in the absence of any commercial or financial relationships that could be construed as a potential conflict of interest.

## Publisher’s Note

All claims expressed in this article are solely those of the authors and do not necessarily represent those of their affiliated organizations, or those of the publisher, the editors and the reviewers. Any product that may be evaluated in this article, or claim that may be made by its manufacturer, is not guaranteed or endorsed by the publisher.

## Abbreviations

ADIPOQ, adiponectin; ADIPOR1, adiponectin receptor 1; ADIPOR2, adiponectin receptor 2; BIA, bioelectrical impedance analysis; BMI, body mass index; CI, confidence interval; CT, computed tomography; DCIS, ductal carcinoma in situ; ER, estrogen receptor; ESA, effective staining area; ESI, effective staining intensity; FFPE, formalin-fixed paraffin-embedded; H&E, hematoxylin and eosin; HER2, human epidermal growth factor receptor 2; LEP, leptin; LEPR, leptin receptor; SD, standard deviation; TMA, tissue microarray; TN, triple negative; WCHS, Women’s Circle of Health Study; WHR, waist-to-hip ratio.
